# A Novel *In Vitro* Method for the Detection and Characterization of Photosensitizers

**DOI:** 10.1371/journal.pone.0015221

**Published:** 2010-12-23

**Authors:** Nadine Karschuk, Yeliz Tepe, Silke Gerlach, Wolfgang Pape, Horst Wenck, Robert Schmucker, Klaus-Peter Wittern, Andreas Schepky, Hendrik Reuter

**Affiliations:** Beiersdorf AG, Research and Development, Hamburg, Germany; Kyushu Institute of Technology, Japan

## Abstract

Photoactivation and binding of photoactive chemicals to proteins is a known prerequisite for the formation of immunogenic photoantigens and the induction of photoallergy. The intensive use of products and the availability of new chemicals, along with an increasing exposure to sun light contribute to the risk of photosensitizing adverse reactions. Dendritic cells (DC) play a pivotal role in the induction of allergic contact dermatitis. Human peripheral blood monocyte derived dendritic cells (PBMDC) were thus perceived as an obvious choice for the development of a novel *in vitro* photosensitization assay using the modulation of cell surface protein expression in response to photosensitizing agents. In this new protocol, known chemicals with photosensitizing, allergenic or non-allergenic potential were pre-incubated with PBMDCs prior to UVA irradiation (1 J/cm^2^). Following a 48 h incubation, the expression of the cell surface molecules CD86, HLA-DR and CD83 was measured by flow cytometry. All tested photosensitizers induced a significant and dose-dependent increase of CD86 expression after irradiation compared to non-irradiated controls. Moreover, the phototoxicity of the chemicals could also be determined. In contrast, (i) CD86 expression was not affected by the chosen irradiation conditions, (ii) increased CD86 expression induced by allergens was independent of irradiation and (iii) no PBMDC activation was observed with the non-allergenic control. The assay proposed here for the evaluation of the photoallergenic potential of chemicals includes the assessment of their allergenic, phototoxic and toxic potential in a single and robust test system and is filling a gap in the *in vitro* photoallergenicity test battery.

## Introduction

Phototoxicity, photoirritancy and photoallergy are topically induced health hazards that can be induced by (simulated) sun light (UV/vis radiation) in the presence of photoreactive agents, generally referred to as “photosensitizers”. The cosmetic and pharmaceutical industries have a particular interest in the identification and evaluation of the photosensitizing potential of new substances before their market launch.

The 7^th^ Amendment to the Cosmetics Directive (Directive 76/768/EEC) aims for the complete replacement of animal testing by 2013. However, due to the public concern regarding the use of animals and the increasing test volume owing to the number of newly developed chemicals the need for innovative *in vitro* alternatives was recognized well before the acceptance of the amendment. Years ago industrial research groups have developed and validated the 3T3 Neutral Red Uptake (NRU) phototoxicity test [1]. These activities resulted in the acceptance by the EU and the OECD acute phototoxicity test guideline No. 432. This guideline test can be performed on fibroblasts or keratinocytes in order to identify the phototoxic potential of a chemical [2]. As an adjunct test the improved protocol of the Photo-red blood cell (RBC) test can be performed as a more mechanistically oriented test system [3]. Reconstructed three dimensional skin models have also been proposed for further evaluation (unpublished prevalidation data). But to date no accepted alternative is available to identify the photoallergenic potential of a new chemical.

Cutaneous photoallergy is understood as a cell mediated delayed hypersensitivity reaction similar to contact allergy where the hapten is a photosensitizer that requires light energy for its activation into a protein reactive compound that may form so called photoantigens and induce an immune response [4].

Briefly, the absorption of (simulated) sun light (incl. UVA radiation) leads to an excitation of a single electron from its ground state to a higher energy level and to the formation of an unstable intermediate or of reactive photometabolites. The activated photohapten may then form a complete photoantigen through covalent binding to a self protein [5]. This photoantigen may then be captured, processed and the derived photohapten-modified peptides presented by dendritic or Langerhans cells to naive T cells in a draining lymph node. This will eventually induce the production of memory and helper T cells, resulting in an antigen-specific immune response (sensitization) [6], [7]. After a further challenge with the same or a cross-reacting photohapten, the immune system will mount an elicitation reaction whose main clinical symptoms are characterized by eczema, papulovesicles, blisters and pruritus. In rare cases ”photosensitization” may generalize beyond the sun exposed area as a persistent light reaction independent from the photosensitizing material [8], [9].

In contrast to the immunologically mediated reaction induced by photoallergens, phototoxic or photoirritant compounds provoke an acute reaction after the first exposure. Upon light exposure, photoirritants generate singlet oxygen and other reactive oxygen species (ROS) that can lead to harmful oxidation of functional cell components and to tissue damage. Moreover, many photosensitizers can induce both phototoxic and photoallergenic reactions [10].

Dendritic cells (DC) play a pivotal role in the initiation and regulation of immune responses. As professional antigen presenting cells they are specialized in the uptake and processing of antigens thereby triggering the complex biological processes leading to specific T cell activation and maturation. During these processes DCs undergo diverse phenotypical and functional changes such as reduced phagocytic ability, upregulated cell surface expression of co-stimulatory molecules and adhesion proteins, such as CD86, CD83, CD54, CD40, and MHC II antigens. In parallel with a modulation of their chemokine receptor pattern, activated and antigen loaded DCs then migrate from the peripheral tissue to draining local lymph nodes [11].

The measurement of these phenotypical and functional DC modifications was central to the development of several *in vitro* test protocols for the detection of sensitizers [12], [13], [14]. Different approaches based on peripheral blood monocyte derived DCs (PBMDCs) or DC-like cell lines such as THP-1, U937 and MUTZ-3 are currently being evaluated and/or validated [15].

We have used DCs derived from peripheral blood primary monocytes to develop an *in vitro* assay for the detection of the photoallergenic potential of chemicals. The modulation of the cell surface molecules CD86, CD83 and HLA-DR was measured after DC exposure to known photoallergenic and phototoxic as well as allergenic and non-allergenic chemicals or to irradiation only. Reproducible and specific results were obtained. Moreover the inter-donor variability of basal CD86 expression was minimal. The test protocol proposed here represents a promising assay for predicting the photoallergenic potential of chemicals including the assessment of their allergenic and toxic/phototoxic potential.

## Materials and Methods

### Chemicals

Chlorpromazine hydrochloride (CPZ, CAS no. 69-09-0), 6-methylcoumarin (6-MC, CAS no. 92-48-8), musk ambrette (MA, CAS no. 83-66-9), sparfloxacin (SPFX, CAS no. 110871-86-8), ketoprofen (KP, CAS no. 22071-15-4) and olaquindox (OLQ, CAS no. 23696-28-8) were applied as known photoallergenic and phototoxic agents. Nickel sulphate (NI, CAS no. 10101-97-0) and α-hexyl cinnamaldehyde (HCA, CAS no. 101-86-0) were used as known allergens and p-hydroxybenzoic acid (HBA, CAS no. 99-96-7) was chosen as negative control (all purchased from Sigma, Germany). Stock solutions of chemicals were prepared in dimethylsulfoxide or ethanol and further diluted in culture medium immediately before use. The final concentration of dimethylsulfoxide or ethanol never exceeded 0.25%.

### Cell culture medium

Culture medium was RPMI 1640 containing 10% heat inactivated (30 min, 56°C) fetal calf serum (FCS Gold, PAA, Austria), 4 mM L-glutamin, penicillin 100 IU/ml, streptomycin 100 µg/ml (all purchased from PAA, Austria).

### Generation of peripheral blood monocyte derived dendritic cells (PBMDC)

Peripheral blood mononuclear cells were isolated from buffy coats of healthy donors by ficoll (Lymphocyte Seperation Medium, PAA, Austria) gradient density centrifugation. Buffy coats were randomly and anonymously obtained as residual product from whole blood preservation production. CD1a^−^/CD14^+^ monocytes were further enriched by positive depletion using anti-CD14-Ig coupled magnetic microbeads (Miltenyi, Germany). PBMDCs were generated by culture of purified monocytes (1×10^6^/ml) in culture medium supplemented with 100 IU/ml of interleukin-4 (IL-4) and 200 IU/ml of granulocyte–macrophage colony-stimulating factor (GM-CSF) for five days as previously described [16].

### Chemical treatment and irradiation of PBMDCs

PBMDCs (7.5×10^5^/ml) were seeded in 24-well plates at day 5 and solutions of test chemicals were added. As a first step, the phototoxicity or cytotoxicity of the chemicals included in the test set was evaluated within a large concentration range limited only by their solubility. The concentration inducing phototoxicity or cytotoxicity around 20% was first determined. The final test range was then defined by choosing a minimum of 4 lower and one higher test concentrations. Irradiation experiments were performed using an UV solar simulator (SOL 500, Dr. Hönle, Germany) equipped with a H1 filter. The H1 filtered spectral output (50% transmission at a wavelength of 335 nm) comprises visible light, UVA and a small amount of UVB (17.3% transmission at 320 nm) and is described elsewhere [1]. The H1 filtered spectrum is later referred to as UV or UVA. The irradiances of UVA light were measured with a calibrated UVA-meter (type No. 37, Dr. Hönle) prior to each experiment. The distance of the UV solar simulator to cell culture plate was 30 cm and irradiation was performed through the cover lid of cell culture plate (Greiner Bio-one, Germany). During the irradiation procedure a ventilator was used to maintain a constant ambient temperature. Chemical treated cells were immediately exposed to simulated sunlight (UVA radiation of 0.5 J/cm^2^ to 4 J/cm^2^) or kept in the dark and then incubated for 48 h at 37°C/5% CO_2_.

### Flow cytometry analysis

Treated PBMDCs were harvested at day 7 (48 h after exposure to the test chemicals) and washed with cold phosphate-buffered saline (PBS). Cells were stained with monoclonal fluorochrome-coupled antibodies (mAb) or with isotype-matched control antibodies. The following mAbs were used: Anti-CD86-APC (clone 2331, FUN-1, lot no. 32914), anti-HLA-DR-APC-Cy7 (clone L243, lot no. 41538) and anti-CD83-FITC (clone HB15e, lot no. 15128). All mAbs were purchased from Becton Dickinson, USA. Dead cells were determined and excluded from further analysis by 7-AAD staining.

PBMDCs were incubated for 15 minutes with mAbs and 10 minutes with 7-AAD. 1×10^4^ cells were analyzed for each sample with a FACS Canto II (Becton Dickinson, USA) and the results were processed by FACS DIVA software (Becton Dickinson, USA).

### Data analysis and statistics

Changes of surface marker expression after treatment (ΔCD86) were calculated relative to untreated controls according to the following formula:
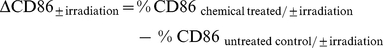



The relative fluorescence intensity (RFI) of HLA-DR was calculated based on the mean fluorescence intensity (MFI) relative to untreated controls according to the following formula: 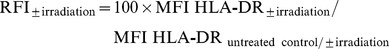



For each test compound data obtained on PBMDCs obtained from at least 5 blood donors were analyzed and are presented as mean values with standard deviation (mean ± SD). When indicated in the figure legends, the significance of the difference between the irradiated ΔCD86 values and the corresponding non-irradiated ΔCD86 values (obtained with test concentrations inducing <20% cytotoxicity) was analyzed using Wilcoxon test, and p-values ≤0.05 were considered to be significant.

## Results

### UV-light sensitivity of PBMDCs

In particular ultraviolet radiation (UVA and UVB) can induce various biological effects including a modulation of the human immune response through phenotypical and functional changes in antigen presenting cells or even functional damage of macrophages, Langerhans or dendritic cells, which can result in an impaired antigen presentation capacity and a suppression of the immune response.

In order to develop an *in vitro* photosensitization assay, irradiation conditions compatible with the envisaged biological test system should first be determined. The effects of two irradiation schemes (measured UVA intensities of 1.7 and 3.4 mW/cm^2^) on PBMDCs were first assessed by exposing the cells to a serial of increasing UVA doses.

**Figure 1:**
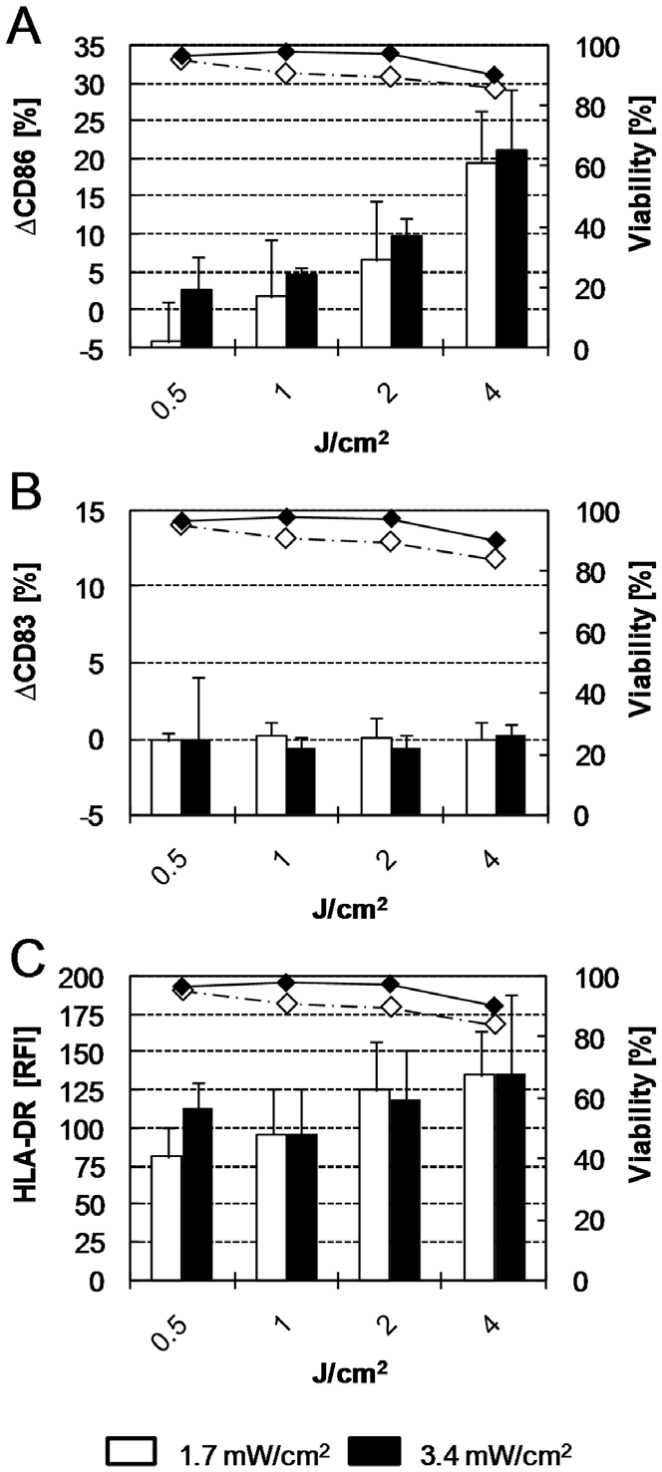
Effects of UVA radiation on the expression of CD86, CD83, HLA-DR and viability of PBMDCs. Cells were exposed to the indicated UVA doses (X axis) with intensities of 1.7 mW/cm^2^ (open columns) and 3.4 mW/cm^2^ (black columns). The expression of CD86 (A), CD83 (B), HLA-DR (C) was analyzed by flow cytometry 48 h later. The corresponding cell viability (right Y axis) is indicated for radiation intensities of 1.7 mW/cm^2^ (open diamonds, dashed line) and 3.4 mW/cm^2^ (black diamonds, solid line). Mean values ± standard deviation of at least 5 independent donors are indicated. RFI: Relative fluorescence intensity.

The viability of PBMDCs irradiated with 0.5 to 2 J/cm^2^ UVA applied with an intensity of 3.4 mW/cm^2^ was not decreased (Fig. 1A, black diamonds). Slight cytotoxic effects (viability decreasing from 97% to 90.3%) were observed when the irradiation dose was increased to 4 J/cm^2^. Similar cytotoxic effects were observed when the same radiation doses were applied with an intensity of 1.7 mW/cm^2^.

The impact of irradiation on the chosen biomarkers (CD86, CD83 and HLA-DR) expression was examined. No relevant increase in CD86 expression could be detected at irradiation doses <1 J/cm^2^. Higher doses induced a dose-dependent increase in CD86 expression (ΔCD86) up to 20% (Fig. 1A, black columns). On the other hand, the expression of the maturation marker CD83 was not affected under these irradiation conditions (Fig. 1B).

With an irradiation intensity of 1.7 mW/cm^2^ the HLA-DR expression first decreased (at 0.5 J/cm^2^) to nearly 75% of the non-irradiated control, and then increased in a dose-dependent manner up to 140% of control (Fig. 1C, white columns). A similar pattern (without initial decrease at 0.5 J/cm^2^) was observed when a radiation intensity of 3.4 mW/cm^2^ was used (Fig. 1C, black columns).

The radiation conditions (dose and intensity) to be used in the photosensitization assay should neither induce cytotoxicity nor relevant alterations in cell surface protein expression but still provide enough energy to activate photosensitizers. Our data indicate that a radiation dose of 1 J/cm^2^ applying an intensity of 3.4 mW/cm^2^ fulfils the first two conditions. These radiation conditions (1 J/cm^2^ UVA with 3.4 mW/cm^2^) were considered as appropriate and used for all further experiments.

### Analysis of inter-donor variability of the basal expression level of the chosen biomarkers in non-irradiated and irradiated PBMDCs

**Figure 2:**
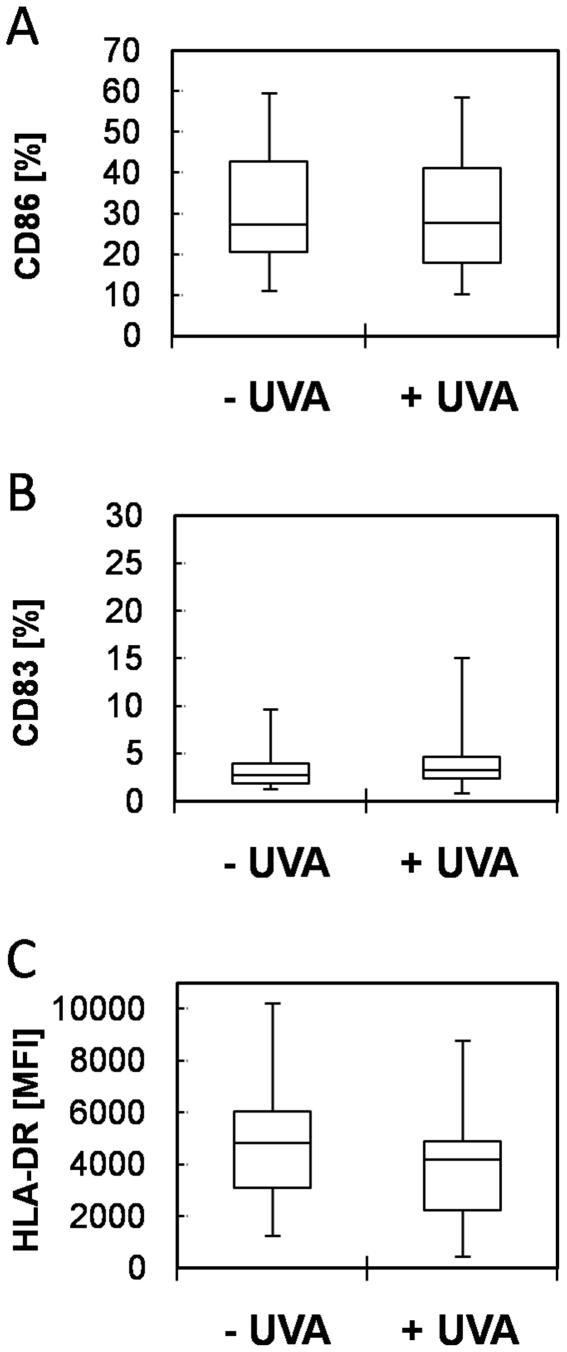
CD86, CD83 and HLA-DR expression of PBMCDs irradiated with 1 J/cm^2^ UVA. The expression of CD86 (A), CD83 (B), HLA-DR (C) was analyzed by flow cytometry on non-irradiated (- UVA) or irradiated (1 J/cm^2^ UVA with 3.4 mW/cm^2^) PBMDCs. Box plots indicate median (horizontal line), 1^st^, 3^rd^ quartile (box) and maxima/minima-values (T-bars) of at least 40 independent donors. MFI: Mean fluorescence intensity.

Donor to donor variability in the basal expression of maturation markers by PBMDCs has been reported in previous studies [14], [17] as a critical point. To evaluate the magnitude of that variability, we investigated the basal CD86, CD83 and HLA-DR surface expression in non-irradiated and irradiated PBMDCs obtained from more than 40 different donors. The CD86 expression level of both control and irradiated cells was relatively low (median values below 30%) with a very limited variance (Fig. 2A). These results were confirmed during a second study performed in our laboratory. The analysis of an extensive data pool over multiple projects, extensive time periods and different laboratory personnel revealed a very stable basal expression of CD86 among donors (n = 397) [18]. Moreover, the overall CD83 expression was low (2.75% without irradiation and 3.3% with irradiation) indicating the immature status of the cells and a very limited variability (Fig. 2B). The HLA-DR expression of the same pool of donors was not influenced by UVA irradiation (as compared to controls, see Fig. 2C) but the extent of donor to donor variability was greater than that observed for CD86 and CD83.

### Induction of CD86 expression in response to photoallergens after irradiation

To evaluate whether this *in vitro* photosensitization assay can detect photosensitizing chemicals using the chosen irradiation conditions (1 J/cm^2^ UVA at 3.4 mW/cm^2^), the following potential photosensitizers were selected and tested: chlorpromazine (CPZ, psychoactive agent), 6-methylcoumarin (6-MC, fragrance), musk ambrette (MA, fragrance), sparfloxacin (SPFX, antibiotic), ketoprofen (KP, nonsteroidal anti-inflammatory drug), olaquindox (OLQ, antibiotic and feed additive). The non-photoallergenic allergens α-hexylcinnamaldehyde (HCA) and nickel sulphate (NI) as well as the non-allergenic 4-hydroxybenzoic acid (HBA) were used as control substances.

**Figure 3:**
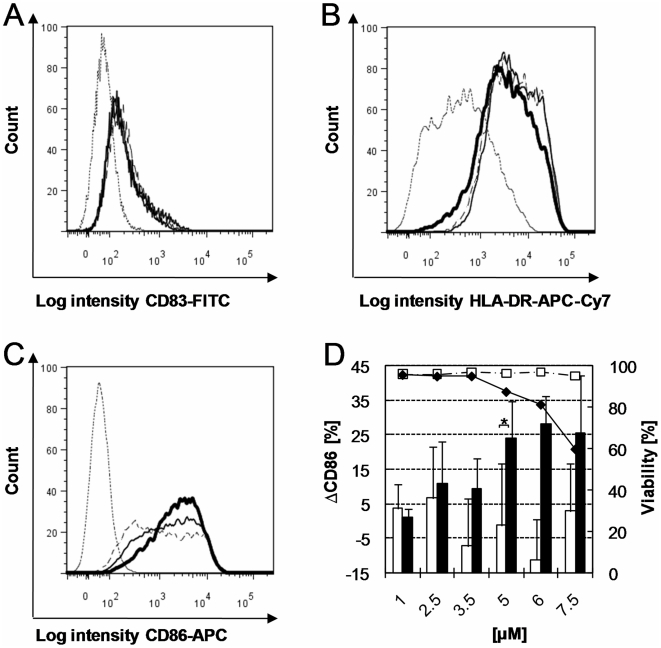
Expression of CD83, HLA-DR, CD86 and viability of PBMDCs exposed to CPZ and UVA radiation. Histogram overlays show CD83 (A), HLA-DR (B) and CD86 expression (C) measured by flow cytometry on irradiated (1 J/cm^2^ with 3.4 mW/cm^2^) PBMDCs. In each histogram the dotted line and thin dashed line indicate the results obtained with the isotype control or untreated control respectively. The results obtained with PBMDCs exposed to CPZ are indicated by a solid line (2.5 µM) or a heavy solid line (5 µM). D: ΔCD86 measured on PBMDCs exposed to the indicated CPZ concentrations (X axis) with (black columns) or without (open columns) UVA radiation (1 J/cm^2^ with 3.4 mW/cm^2^). The viability (right Y axis) of non-irradiated (open squares, dashed line) and irradiated cells (black diamonds, solid line) is also indicated. Mean values for cell viability and mean ± standard deviation for ΔCD86 expression of at least 5 independent donors are shown. Negative ΔCD86 values were due to biological variability. Asterisk indicates significant difference between irradiated and non-irradiated ΔCD86 values (p<0.05, Wilcoxon test).

PBMDCs were first exposed to increasing concentrations of CPZ (known photosensitizer with photoallergenic and phototoxic properties) with and without irradiation. CD86, CD83 and HLA-DR surface expression of photosensitizer treated, non-irradiated as well as photosensitizer treated, irradiated cells and controls were compared (Fig. 3). Minor or irrelevant modifications in CD83 expression were observed (CPZ treated, irradiated cells versus irradiated control, Fig. 3A) and only a slight increase in HLA-DR expression was induced by CPZ under irradiation (Fig. 3B). In contrast, a marked increase in CD86 expression was observed under those same conditions (Fig. 3C). CD86 modulation calculated as the difference between the results obtained for the treated sample and the corresponding untreated control (ΔCD86) revealed a dose-effect relationship in response to CPZ after irradiation (Fig. 3D, black columns). Increasing concentrations from 2.5 µM to 5 µM induced a 2.2-fold increase of ΔCD86 (11% to 24%). This represents a very significant increase in CD86 expression compared to the low ΔCD86 values obtained without irradiation at 5 µM. Negative ΔCD86 values were due to biological variability. Moreover CPZ treatment induced dose-related cytotoxic effects with irradiation but had no cytotoxic effect without irradiation.

Since CD86 was markedly upregulated in response to irradiated CPZ (in contrast to CD83 and HLA-DR), it was chosen as the sole marker for the analysis of the following experiments.

**Figure 4:**
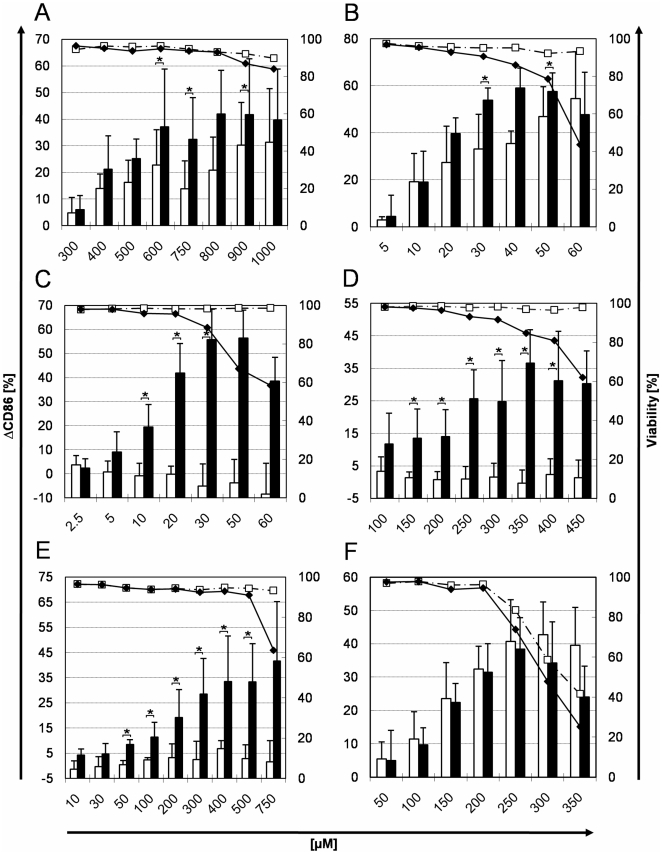
CD86 expression and viability of PBMDCs exposed to photoallergens and allergens with and without irradiation. PBMDCs were incubated with the indicated (X axis) concentrations of photoallergens and allergens with or without exposure to 1 J/cm^2^ UVA. The ΔCD86 values (columns) and the cell viability (lines) were measured by flow cytometry without (open columns/squares) or with UVA irradiation (black columns/diamonds) after exposure to the following compounds: A: 6-methylcoumarin. B: musk ambrette. C: sparfloxacin. D: ketoprofen. E: olaquindox. F: α-hexylcinnamaldehyde. Mean values for cell viability and mean ± standard deviation for the ΔCD86 expression values measured on the PBMCDs from at least 5 independent donors are shown. Negative ΔCD86 values were due to biological variability. Asterisk indicates significant difference between irradiated and non-irradiated ΔCD86 values (p<0.05, Wilcoxon test).

6-MC (responsible for photocontact dermatitis reactions) induced a dose-dependent increase in CD86 expression after irradiation (>40% ΔCD86, Fig. 4A). The difference with the ΔCD86 obtained without irradiation was significant at concentrations >600 µM.

6-MC without irradiation induced a ΔCD86 increase >20% compared to the untreated control. No cytotoxic effects were detected within the tested concentration range without irradiation. Under irradiation, a slight cytotoxicity (16%) was detected at the highest test concentration.

MA, a known allergenic and photoallergenic fragrance [19] was tested with PBMDCs isolated from 6 different donors. A dose-related increase in CD86 expression (ΔCD86) was observed with or without irradiation, the increase being nevertheless significantly higher after irradiation (Fig. 4B). ΔCD86 values exceeded 50% at 30 µM whereas cell viability decreased below 80% at 50 µM under irradiation. On the other hand, non-irradiated MA had no cytotoxic effect over the whole tested concentration range.

SPFX, an extreme phototoxic chemical with weak photoallergenic properties [20] induced a significant ΔCD86 increase (Fig. 4C) under irradiation conditions compared to non-irradiated cells. At low concentrations (≤10 µM) it already induced a ΔCD86 level >20% without cytotoxic effect. At higher doses with irradiation, it was relatively cytotoxic (>30% cytotoxicity at 50 µM). In contrast, without irradiation, no relevant cytotoxicity was detected.

Exposure to KP, a photoallergenic nonsteroidal anti-inflammatory drug, induced after irradiation a dose-dependent increase of ΔCD86 >25% without cytotoxic effects up to 300 µM (Fig. 4D, black columns and black diamonds). At high doses (>350 µM), irradiated KP induced phototoxic effects. On the other hand, KP alone (without irradiation) had no relevant effect on the CD86 expression or on the cell viability.

OLQ, a photoallergenic feed additive and antibiotic, induced under radiation conditions a dose-related increase in CD86 expression (ΔCD86) values >30% at concentrations >400 µM (Fig. 4E, black columns) and cytotoxicity was detected at the highest test concentration only (750 µM). In contrast, non-irradiated OLQ treatment resulted in limited CD86 modulation (ΔCD86<10%) and did not influence cell viability.

To determine whether the described photosensitization assay is able to distinguish photosensitizers from allergens and non-allergens, non-photosensitizing allergens (for example HCA and NI) were used as controls. As expected, the fragrance compound HCA induced relevant and analogous increases of CD86 expression with or without irradiation (ΔCD86±UV >30% at 200 µM, see Fig. 4F). The cytotoxic effects were similarly induced at concentrations >200 µM under both conditions.

**Figure 5:**
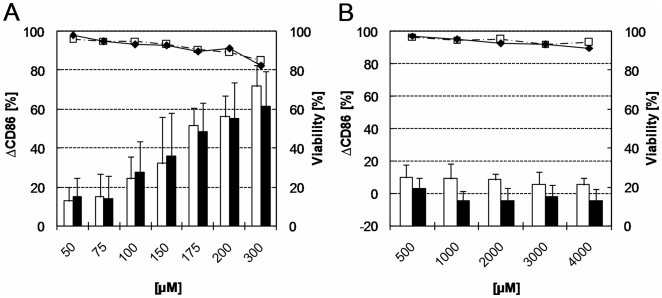
CD86 expression and viability of PBMDCs exposed to nickel sulphate and p-hydroxybenzoic acid. PBMDCs were incubated with the indicated (X axis) concentrations of nickel sulphate (A) and p-hydroxybenzoic acid (B) with or without exposure to 1 J/cm^2^ UVA. The ΔCD86 values (columns) and the cell viability (lines) were measured by flow cytometry without (open columns/squares) or with UVA irradiation (black columns/diamonds) after exposure to the test compounds. Mean values for cell viability and mean ± standard deviation for the ΔCD86 values measured on the PBMCDs from at least 5 independent donors are shown. Negative ΔCD86 values were due to biological variability. Asterisk indicates significant difference between irradiated and non-irradiated ΔCD86 values (p<0.05, Wilcoxon test).

Likewise, NI induced very similar dose-dependent increases of the CD86 expression with or without irradiation (ΔCD86>55% at 200 µM, see Fig. 5A). The radiation condition had no impact on the relatively weak NI induced cytotoxicity observed at 300 µM.

HBA, a non-allergenic and non-photoallergenic compound was chosen as negative control. Cell viability and the modulation of CD86 expression (ΔCD86) in response to 500 µM–4 mM HBA with or without irradiation are shown in Fig. 5B. As expected, no relevant modulation of the CD86 expression (ΔCD86<10%) was measured with or without irradiation and no cytotoxic effect was observed up to the highest test concentration (4 mM).

## Discussion

The objective of this study was to develop and evaluate an *in vitro* model for the detection and characterization of the photoallergenic potential of chemicals. The *in vitro* approach presented here uses the modulation of certain proteins expressed on the surface of PBMDCs exposed to photoallergens with and without irradiation as a readout system.

First, radiation conditions compatible with the envisaged photosensitization assay had to be defined. The applied UVA dose should have no relevant impact on the chosen surface markers (CD86, HLA-DR and CD83) as well as on the viability of the PBMDCs. Initially, the radiation conditions proposed for the 3T3 NRU phototoxicity test [2] were adapted to our system. Dose-dependent increases in CD86 and HLA-DR (but not CD83) expression were observed at radiation doses >1 J/cm^2^ UVA. A 10–15% cytotoxicity was induced at 4 J/cm^2^ (UV induced apoptosis, see Timares et al. [21]). This is similar to the results published by Mittelbrunn et al. [22] who reported a partial maturation of PDMDCs and approx. 33% cytotoxicity after irradiation with 5 J/cm^2^ UVA and 0.4 J/cm^2^ UVB generated by a xenon solar simulator.

In contrast, a radiation dose of 1 J/cm^2^ had no effect on the three chosen activation markers or on the PBMDCs viability. These radiation conditions (1 J/cm^2^ UVA, 3.4 mW/cm^2^) were considered as appropriate and used for all further experiments.

Donor to donor variability in the basal expression of maturation markers by PBMDCs has been reported as a critical point in previous studies [14], [17], [23], [24] and was often considered as an obstacle to the use of PBMDCs for the development of *in vitro* sensitization tests and DC-like cell lines were perceived as better alternatives [15]. The magnitude of that variability was investigated by measuring the expression of CD86, CD83 and HLA-DR on non-irradiated and irradiated, immature PBMDCs. Their expression levels were shown to be very similar in cells isolated from a large set of different donors. This limited donor variability was already published by Coutant *et al.* [25] and has been extensively studied and recently confirmed by our group (n = 397; median CD86 expression 26.7%, 1^st^ quartile 17.7%, 3^rd^ quartile 40.5%) [18]. However, an intrinsic variability of primary cells is to be expected due to the diversity in individual immune responses to allergens in the population. In order to take advantage of this existing but limited variability, we decided to test the photoallergenic potential of a chemical with PBMDCs obtained from a minimum of 5 independent donors.

CPZ, a known photosensitizer with photoallergenic and phototoxic properties, was tested with and without irradiation and the induced modulation of CD86, CD83 and HLA-DR expressions as well as cell viability were measured by flow cytometry and compared 48 h after treatment. Only minor modifications of HLA-DR and CD83 expressions could be detected. In contrast, CD86 measurements revealed a significant dose-effect relationship in response to CPZ after irradiation. Moreover, CPZ treatment induced dose-related cytotoxic effects with irradiation but had no cytotoxic effect without irradiation. CD86 induction was thus perceived as a suitable endpoint for photoallergen detection. This is consistent with the numerous reports indicating CD86 as one of the most promising amongst frequently tested DC activation markers in skin sensitization testing [15].

Further chemicals with documented photoallergenic and phototoxic potentials were used to evaluate the performance of the proposed *in vitro* photosensitization assay. The ΔCD86 values induced by the photoallergens after irradiation were always more than 15% higher than the corresponding values obtained without irradiation. This difference (15%) between the ΔCD86 values measured with and without irradiation could be used as a putative cut off value for detecting photoallergens. As an alternative, a statistically significant difference between the ΔCD86 values obtained with or without irradiation might be considered. Further tests with an extensive set of chemicals will be necessary to get a robust cut off value.

When testing CPZ it was observed that the induction of CD86 expression appeared at doses close to or overlapping with those inducing cytotoxic effects as a result of both photoallergenic and phototoxic reactivity. In another study, we could demonstrate that the presence of 33% dead cells induced the unspecific upregulation of the CD86 expression [18]. These unspecific cytotoxic effects due to damaged and/or dead cells have to be excluded and we propose that data obtained with test concentrations inducing >20% cytotoxicity should not be considered for the determination of photoallergenicity.

Using our assay, MA a known allergenic and photoallergenic fragrance [19], [26] induced a dose-related increase in CD86 expression with or without irradiation, the increase being significantly higher after irradiation. We could thus classify MA as an allergen and a photoallergen with phototoxic properties. This is consistent with available *in vivo* data indicating that contact dermatitis to MA can be exacerbated by irradiation [19], [27]. Moreover, the validated 3T3 NRU phototoxicity test also classified MA as phototoxic [28].

The fragrance 6-MC was detected as a potent photoallergen consistent with other *in vivo* and *in vitro* data indicating a role of photodimerized 6-MC esters in 6-MC photoallergenicity [29], [30]. Using our protocol, the highest test concentration induced a slight phototoxic effect suggesting a pronounced cytotoxicity at concentrations >1 mM. Though cytotoxicity of irradiated 6-MC was also detected in the THP-1 cell line based *in vitro* approach described by Hoya et al. [31] and its phototoxicity partially attributed to the formation of singlet oxygen [32], it was concluded that 6-MC is a photoallergen only. Here, non-irradiated 6-MC also resulted in a relevant CD86 induction indicating an allergenic potential consistent with Buehler et al. [33] who considered 6-MC as contact allergen with weak phototoxic potential.

An inverse correlation between phototoxic effects and photoallergenicity was demonstrated for some flouroquinolone antibiotics. For example, SPFX is known as an extreme phototoxic chemical with weak photoallergenic properties [20]. Its weak photoallergenicity is likely outweighed by its strong phototoxicity, e.g. when high concentrations are applied in medical formulations. In our test, irradiated SPFX induced a relevant CD86 modulation already at low concentrations (≤10 µM) without cytotoxic effect, thus indicating a photoallergenic potential. Interestingly, photohaptenic properties were also described by Hino et al. [34] where SPFX treatment of THP-1 cells only resulted in enhanced HLA-DR but not CD86 or CD54 expression.

In recent years, increasing incidences of photoallergic reactions to non-steroidal anti-inflammatory drugs, mainly KP, were reported. Our *in vitro* test clearly classified KP as a photoallergen and phototoxin consistent with previous *in vivo* and *in vitro* studies [35]. KP is also known to elicit contact dermatitis [36]. Since allergenic and photoallergenic concentrations are not necessarily identical, we hypothesized that higher KP concentrations would be required in our assay to reveal KP's allergenic potential (without irradiation) than its photoallergenic potential. Indeed, KP induced a dose-dependent increase of CD86 expression after irradiation whereas without irradiation it had no relevant effect on the CD86 expression or on the cell viability.

Using our test protocol, the photoallergenic feed additive OLQ induced significant and dose-related increases in CD86 expression and cytotoxicity was detected at the highest test concentration. In contrast, non-irradiated OLQ treatment resulted in limited CD86 modulation and did not influence cell viability. Since the ΔCD86 values obtained with or without irradiation showed a difference of more than 30% our results are consistent with the photoallergenic and phototoxic properties deduced from other studies [9], [37].

HCA demonstrated a very similar and dose-dependent CD86 induction with and without irradiation and we thus concluded that HCA has no photoallergenic but an allergenic potential. HCA was classified as a contact sensitizer serving as a positive control in the LLNA and tested in other *in vitro* sensitizations tests [15], [38]. The cytotoxicity was also similar under both conditions indicating only toxic (but no phototoxic) properties at high concentrations. This result is also consistent with data obtained from a photohaemolysis test [39].

NI induced similar dose-dependent increases of the CD86 expression under both conditions and irradiation had no influence on its relatively weak cytotoxicity. This is the reaction pattern expected for non-photosensitizing allergens such as NI (and HCA) and our *in vitro* results were in complete agreement with its clinical relevance (induction of allergic contact dermatitis) or its use as a model for metal allergens [40], [41], [42].

HBA was tested as a typical non-allergenic and non-photoallergenic compound. As expected, no relevant modulation of the CD86 expression was detected with or without irradiation and no cytotoxic effect was observed up to the highest test concentration. These results are again in full agreement with already published *in vitro* tests and LLNA study results [43], [44].

All tested chemicals possessing a photoallergenic potential could be clearly distinguished from allergens. Chemicals with a photoallergenic potential also showed phototoxic effects at higher concentration ranges that could be clearly determined in our *in vitro* assay. However, it may not be excluded that highly phototoxic drugs are able to induce additional photoallergic reactions since many drugs are able to bind to biomolecules after activation. This consideration is constantly supported by reports describing photoallergic reactions induced by drugs considered as photoirritants. However, the chemical process leading to protein photobinding should be intensively investigated since our present understanding is insufficient for a clear distinction of photoallergens and photoirritants *in vitro* [45]. In addition photopatch tests do not lead to an unambiguous clinical determination of photoallergic and phototoxic reactions since multiple factors may influence dermal responses to chemicals, e.g. pre-existing skin disorders or cross reactions [46].

Due to the 7^th^ Amendment of the European Cosmetic Directive, the cosmetic industry needs to finalize the development of alternatives to animal tests by 2013. *In vitro* assays have already been developed for acute phototoxicity testing and the OECD proposes to use the validated 3T3 NRU phototoxicity test which can be performed on fibroblasts or keratinocytes [47]. However, these cell types play different roles in the induction of photoallergy and a DC based assay would represent a critical and major addition to the available test panel.

In summary, this novel *in vitro* photosensitization assay based on primary PBMDCs yields reproducible and specific results allowing a precise determination of the photosensitizing characteristics of the tested compounds. The described assay represents a promising and robust test system for predicting the photoallergenic potential of chemicals including the assessment of their allergenic and toxic/phototoxic potential and is filling a gap in the *in vitro* photoallergenicity test battery.
